# miRNA-130a-3p targets sphingosine-1-phosphate receptor 1 to activate the microglial and astrocytes and to promote neural injury under the high glucose condition

**DOI:** 10.1515/med-2022-0565

**Published:** 2022-12-20

**Authors:** Guang Yang, Jinxin Shi

**Affiliations:** Department of Pain, Funing County People’s Hospital, Funing County, Yancheng City, Jiangsu Province, 224400, China; Department of Pain, Funing County People’s Hospital, No 111 Fucheng Street, Funing County, Yancheng City, Jiangsu Province, 224400, China

**Keywords:** miR-130a-3p, S1PR1, microglial and astrocyte activation, neural injury, high glucose

## Abstract

As a common complication of diabetes, diabetic pain neuropathy (DPN) is caused by neuron intrinsic and extrinsic factors. Neuron intrinsic factors include neuronal apoptosis and oxidative stress, while extrinsic factors are associated with glial activation. The present study was performed to reveal the functions of miR-130a-3p in apoptosis and oxidative stress of the high glucose (HG)-stimulated primary neurons as well as in the activation of microglial and astrocytes. Primary neurons, microglial, and astrocytes were isolated from newborn mice. Apoptosis was assessed by flow cytometry analysis and western blotting. Reactive oxygen species and glutathione levels were assessed to determine the oxidative stress. Markers of glial cells were detected by immunofluorescence staining. The results revealed that miR-130a-3p deficiency alleviated apoptosis and oxidative stress of HG-stimulated neurons as well as suppressed microglial and astrocyte activation. Moreover, sphingosine-1-phosphate receptor 1 (S1PR1) was found as a target downstream of miR-130a-3p. S1PR1 knockdown partially rescued the inhibitory effects of silenced miR-130a-3p on neuronal injury and glial activation. In conclusion, miR-130a-3p targets S1PR1 to activate the microglial and astrocytes and to promote apoptosis and oxidative stress of the HG-stimulated primary neurons. These findings may provide a novel insight into DPN treatment.

## 1 Introduction

Diabetic pain neuropathy (DPN) refers to symptoms of dysfunctions of peripheral nerves in diabetic people after excluding other causes [1]. DPN is the most common risk factor for neuropathy [2] and leads to high mortality, morbidity, and amputation in patients with diabetes [3,4]. The causes of DPN include neuron intrinsic and extrinsic factors. Current theories on neuron intrinsic factors indicated that uncontrolled oxidative stress drives the development of DPN [5], while studies on extrinsic factors revealed that the neuro-immune interaction between activated pain-sensing neurons and microglia controls DPN [6–8].

The competence and integrity of the functional neurons are associated with the interactive exchanges among glial cells, interneurons, and sensory and motor neurons [9]. Astrocytes and microglia are residents in the central nervous system. Microglia regulates the neuroinflammation in DPN [10,11]. M1 microglia can induce the release of proinflammatory factors (e.g., TNF-α, IL-6, IL-1β), chemokines, reactive oxygen species (ROS), and nitric oxide [12–15]. Astrocytes supply neurons with trophic support, contribute to the formation and pruning of synapses, and maintain some homeostatic functions [16]. Induced by microglia, A1 astrocytes lose most functions of normal astrocyte and becomes neurotoxic to rapidly kill neurons and maturely differentiated oligodendrocytes [17]. A1 astrocytes are activated in many neurodegenerative diseases, including Parkinson’s disease, Huntington’s disease, multiple sclerosis, and Alzheimer’s disease [17,18]. Thus, inactivating the A1 astrocytes and M1 microglia as well as inhibiting the excessive neuroinflammation are promising for DPN treatment.

Increasing evidence suggests that genetic factors regulate DPN pathogenesis [19]. MicroRNAs (miRNAs) are short, single-stranded non-coding RNAs with 18–22 nucleotides in length, post-transcriptionally regulating mRNA expression by binding to the 3′-untranslated region (3′-UTR) of their target genes [20]. Multiple types of research have revealed the significant function of miRNAs in microglial activation [21], astrocyte activation [22] as well as in oxidative stress of neurons [23] in pain-related diseases. Moreover, miRNAs can serve as biomarkers for DPN [24].

MiR-130a-3p promotes spinal cord injury-induced neuropathic pain (NP) by inducing neuronal apoptosis and microglial activation in rats [25]. We inferred that miR-130a-3p also participates in DPN and its function is related to neuronal injury, microglial activation, and astrocyte activation. We used the high glucose (HG)-stimulated primary neurons to mimic the diabetes-induced neuronal injury *in vitro*, and detected the impacts of miR-130a-3p deficiency on the apoptosis and oxidative stress of neurons. Effects of miR-130a-3p deficiency on activation of microglia and astrocytes were evaluated followed by the investigation of its downstream targets.

## 2 Materials and methods

### 2.1 Patient sample collection

A total of 30 DPN patients and 30 volunteers with lower limb skin samples were collected in this study. All samples were cut into small pieces and stored at −80°C.


**Ethical approval:** Written consent was obtained from all patients, and the study was approved by the ethics committee of the Funing County People s hospital.

### 2.2 Culture of primary microglia

Microglial cells were isolated from 1-day-old C57BL/N mice as previously reported [26]. In brief, 0.5–1 mm^3^ pieces of brain tissues were treated with 0.25% trypsin–EDTA (Kaissy, SH-2464) solution for 10 min. Tissues were digested by trypsinization and centrifuged at 300×*g* for 5 min followed by resuspension in DMEM/F12 (Gibco, C11330500BT). After filtration using a 100-μm nylon mesh, the cell suspension was cultured in poly-l-lysine pre-coated T75 flasks. The cell culture medium (DMEM/F12) was changed every 5 days, and after 14 days of culture, the mixed glial cultures were shaken at 220*g* for 2 h at 37°C to facilitate detachment of microglia from the flask. Cell culture medium containing released microglia was collected from the flask and cells were collected by centrifugation at 1,000×*g* for 5 min. Cell supernatants were cultured in poly-l-lysine pre-coated 24-well plates at 37°C in a humified environment with 5% CO_2_. Every 3 days, the medium was changed. Lipopolysaccharide (LPS; 1 μg/mL; Sigma-Aldrich, L2630-100mg) was used to stimulate the microglia for 1 day to stimulate the inflammatory phenotype [27]. The medium from LPS-treated microglial was harvested as a microglia-conditioned medium (MCM).


**Ethics approval and consent to participate:** The experimental protocol was established, according to the ethical guidelines of the Helsinki Declaration. The animal experiments were conducted based on the approved animal protocols and guidelines established by Funing County People’s Hospital.

### 2.3 Culture of primary astrocyte

After *in vitro* culture for 3 days, confluent cultures were centrifugated at 200 rpm for 120 min to remove the microglia and were subsequently cultured in the astrocyte culture medium followed by centrifugation for removal of oligodendrocyte precursor cells. MCM was used to treat the remaining astrocytes for 24 h to produce activated astrocytes.

### 2.4 Culture of primary neurons

Cortical tissue from 1-day-old C57BL/N mice was collected and minced. Digested with 0.25% trypsin-EDTA for 30 min at 37°C. The samples were then eluted with 10% fetal bovine serum. Then, centrifugation (1,500 rpm, 5 min) was performed at room temperature. The supernatant was discarded after centrifugation, and a single-cell suspension was prepared with a fresh complete medium. Subsequently, cells were seeded with about 1.5 × 10^5^ cells/cm^2^ density on poly-l-lysine-coated (100 mg/L) 12-well plates. Neuron-specific medium (contained neurobasal medium; Thermo Fisher Scientific), 2 mM glutamine, 2% B27 neurobasal supplement (Gibco), 1% penicillin–streptomycin, and 1% 100× GlutaMAX) were added and incubated at 37°C with 5% CO_2_ and 95% air. The medium was changed every 3 days.

### 2.5 Cell treatment

Microglial cells, astrocytes, and neurons were stimulated with HG (30 mM) for 24 h [28]. Cells were transfected with NC inhibitor, miR-130a-3p inhibitor, or cotransfected with miR-130a-3p inhibitor + si-S1PR1 by Lipofectamine 3000 (Thermo Fisher Scientific) for 48 h at room temperature. NC mimics, miR-130a-3p mimics, NC inhibitor, miR-130a-3p inhibitor, si-NC, and si-S1PR1 were purchased from GenePharma (Shanghai, China).

### 2.6 Reverse transcription quantitative polymerase chain reaction (RT-qPCR)

Total RNA was extracted from neurons using the TRIzol^®^ reagent (Invitrogen, Carlsbad, CA, USA, 15596-018). Complementary DNA was synthesized using a reverse transcription system (Toyobo, Osaka, Japan). PCR was performed on an ABI 7900 fast real-time PCR system with SYBR Green PCR master mix (both from Applied Biosystems). Expression levels of miR-130a-3p and its potential targets were normalized to the internal references U6 and GAPDH, respectively, and were calculated by the 2^−ΔΔCT^ method. Primers were designed using the NCBI Primer-Blast (https://www.ncbi.nlm.nih.gov/tools/primer-blast/index.cgi?LINK_LOC=BlastHome). Primer sequences were listed as follows:

miR-130a-3p, F: 5′-CAGTGCAATGTTAAAAGGGCA-3′;

R: 5′-CTCTACAGCTATATTGCCAGCCAC-3′.

S1PR1, F: 5′- CCTATTAGCAGGCGTGGCTT-3′;

R: 5′- TTCCATGCCTGGGATGATGG-3′.

GAPDH, F: 5′-CATCTTCTTGTGCAGTGCC-3′;

R: 5′-CAAATCCGTTCACACCGAC-3′.

U6,F: 5′-CTCGCTTCGGCAGCACA-3′;

R: 5′-ACGCTTCACGAATTTGCGT-3′.

### 2.7 Western blotting

Proteins were extracted from neurons, astrocytes, and microglial cells using the RIPA lysis buffer and were quantified using the BCA method. After separation by SDS-PAGE, proteins (20 μg/lane) were transferred to PVDF membranes (Millipore, Burlington, MA, USA, IEVH00005) and blocked with 5% bovine serum albumin (v/v). Next, membranes were incubated overnight at 4°C with primary antibodies including cleaved caspase 3 (1/5,000, ab214430; Abcam), cleaved caspase 9 (1/1,000, #9509; Cell Signaling Technology), iNOS (1/1,000, ab178945; Abcam), C3 (1/2,000, ab97462; Abcam), and β-actin (1/2,000, ab8226; Abcam) followed by incubation with the secondary antibody IgG at room temperature for 2 h. Protein bands were visualized using the ECL reagent (Thermo Fisher Scientific) and semi-quantified using the ImageJ software (National Institutes of Health).

### 2.8 Cell viability assay

Cell viability was assessed by a cell-counting kit 8 (CCK-8; DoJindo, Japan, ck04) according to the manufacturer’s protocols. Cells were plated into six-well plates with 2 mL of complete medium and were incubated with CCK-8 solution for 24, 48, and 72 h. Optical density (OD) was detected by a microplate reader (Bio-Rad, Hercules, CA, USA) at the absorbance of 450 nm.

### 2.9 Flow cytometry apoptosis analysis

Cell apoptotic rates were assessed by Annexin V-FITC/PI double staining assay (BD Biosciences, 556547) based on the manufacturer’s protocol. Cells were stained with 10 μL of PI dye and 5 μL of Annexin V dye for 15 min at room temperature. Apoptosis was analyzed using the BD Accuri^TM^ C6 Plus flow cytometry. Cells in the second and third quadrants were considered apoptotic.

### 2.10 ELISA

Supernatants of microglial cells were collected to measure the concentrations of TNF-α, IL-1α, IL-1β, and IL-6 using corresponding ELISA kits (ab208348, ab113344, ab197742, ab222503) according to the manufacturer’s protocols. OD was measured with a plate reader by detecting absorbance at 450 nm.

### 2.11 Immunofluorescence staining

Cells were fixed in 4% paraformaldehyde for 30 min and treated with 0.1% Triton X-100 and 10% goat serum for 60 min at room temperature for blocking. Next, microglial cells were incubated with primary antibodies against IBA1 (1/500, ab178846) and INOS (1/500, ab178945), while astrocytes were incubated with primary antibodies against GFAP (1/300, #3670; Cell Signaling Technology) and C3 (1/100, ab97462) overnight at 4°C. On the next day, microglial cells and astrocytes were incubated with fluorescence-labeled secondary antibodies. Nuclei were counterstained with DAPI. A fluorescence microscope (AxioVertA1 and ImagerA2) was used to capture the fluorescent images.

### 2.12 Evaluation of ROS and glutathione (GSH)

The supernatants of neurons were collected. Intracellular ROS were detected by the 2′,7′-dichlorodihydrofluorescein diacetate (H2DCF-DA) using a DCFDA/H2DCFDA-Cellular ROS Assay Kit (ab113851) according to the manufacturer’s guidelines. GSH estimation was performed according to a previous study [28]. Images were photographed by a confocal microscope. Data were quantified using ImageJ software.

### 2.13 Immunocytochemistry assay

Neurons were fixed with 4% formaldehyde for 2 h, permeabilized with 2% Tween 20, washed with PBS, and blocked in 5% BSA for 30 min. Next, cells were incubated with the Nav1.8 antibody (1:200, ab93616) overnight. After washing, cells were incubated with the FITC-conjugated secondary antibody IgG in an antiserum diluent. Photos were taken using a Confocal Laser Scanning Microscope [29].

### 2.14 Luciferase reporter assay

Wild-type (WT) or mutated (MUT) sequences of S1PR1 3′-UTR complementary to miR-130a-3p were synthesized by GeneScript (Nanjing, China) and were subcloned into the FseI and XbaI restriction sites of the pGL3 luciferase reporter vector (Promega, Madison, WI, USA) to generate the S1PR1 3′-UTR WT or S1PR1 3′-UTR MUT reporter constructs. Cells were transfected with miR-124-3p mimics, or NC mimics were plated into 96-well plates and were cotransfected with 100 ng of S1PR1 3′-UTR WT or S1PR1 3′-UTR MUT. Firefly luciferase signals were normalized to *Renilla* luciferase signals using a Dual-Luciferase^®^ Assay Kit (Promega, E1910).

### 2.15 RNA pull-down assay

RNA pull-down assays were performed using a Pierce^TM^ Magnetic RNA Protein Pull-Down kit (Thermo Fisher Scientific). Briefly, S1PR1-WT, S1PR1-Mut, and NC were biotin labeled with biotin for S1PR1-WT, S1PR1-Mut, and NC, respectively. Then, cells were lysed and cultured with biotinylated probes and M-280 streptavidin magnetic beads (Invitrogen, USA). Finally, RT-qPCR was performed to detect the expression of miR-124-3p.

### 2.16 Statistical analyses

Data derived from three independent biological replicates were presented as mean  ±  standard deviation. SPSS 25.0 was used for statistical analysis. A Student’s *t*-test was utilized for different comparisons between two groups. One-way ANOVA was utilized for comparing differences among multiple groups. A *P*-value less than 0.05 was significant on a statistical basis.

## 3 Results

### 3.1 MiR-130a-3p expression in DPN patients and HG-stimulated astrocytes, neurons, and microglial cells

**Figure 1:**
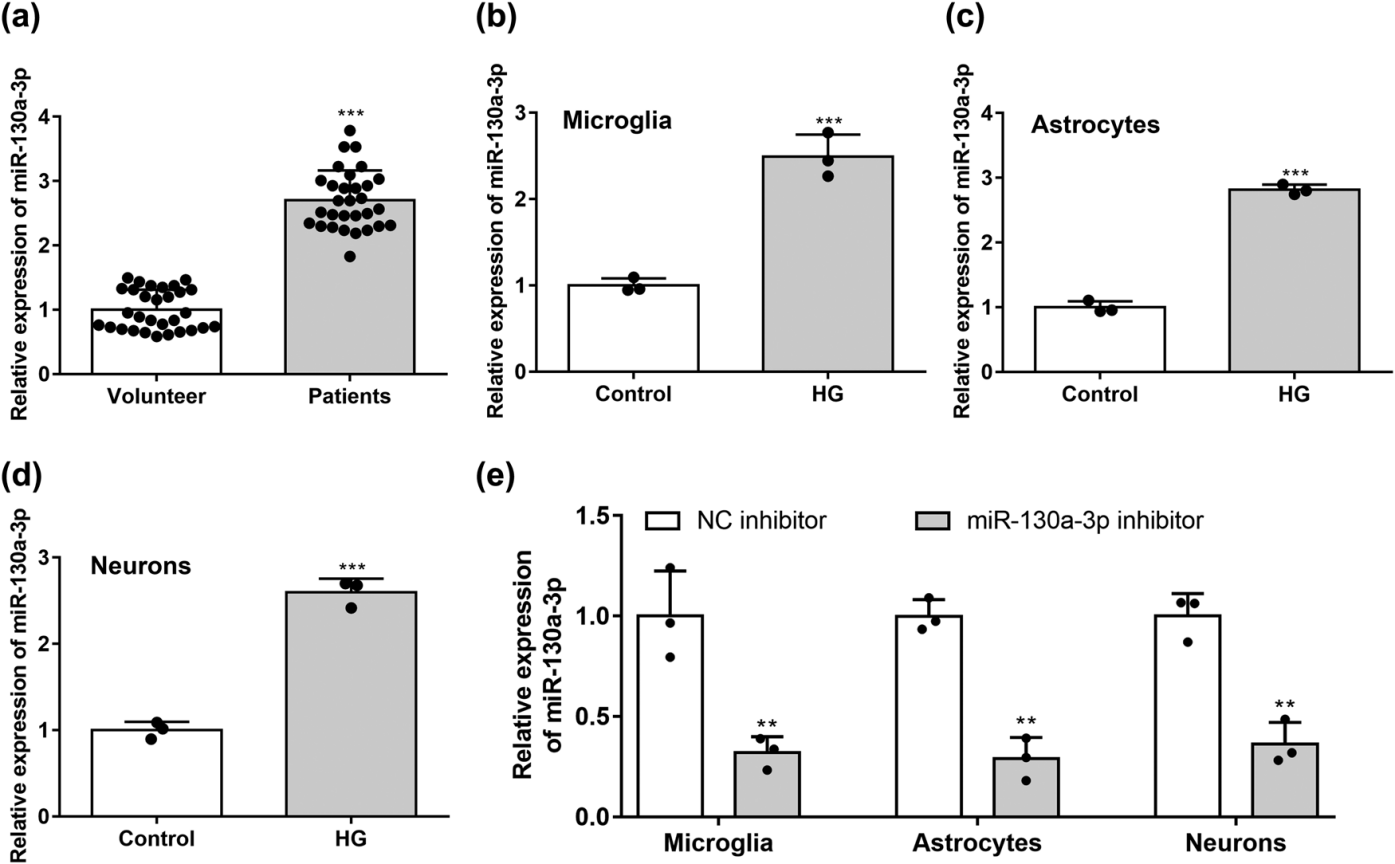
Expression of miR-130a-3p in DPN patients and HG-stimulated microglia, astrocytes, and neurons. (a) Expression of miR-130a-3p in DPN patients’ tissues was determined by RT-qPCR. Expression of miR-130a-3p in HG-stimulated microglia (b), astrocytes (c), and neurons (d) compared to that in basal cells was evaluated by RT-qPCR. (e) Expression of miR-130a-3p in HG-stimulated microglia, astrocytes, and neurons by RT-qPCR. ***P < 0.001 vs control.

MiR-130a-3p was found to be highly expressed in the lower limb skin tissues of DPN patients according to RT-qPCR assays (Figure 1a). Figure 1b–d revealed that HG induced the upregulation of miR-130a-3p in isolated microglial cells (Figure 1b), astrocytes (Figure 1c), and neurons (Figure 1d). Figure 1e shows that the expression of miR-130a-3p in microglia, astrocytes, and neurons was significantly reduced after transfection with miR-130a-3p inhibitor.

### 3.2 MiR-130a-3p inhibitor promoted viability and suppressed apoptosis of microglial cells, astrocytes, and neurons under the HG condition

**Figure 2:**
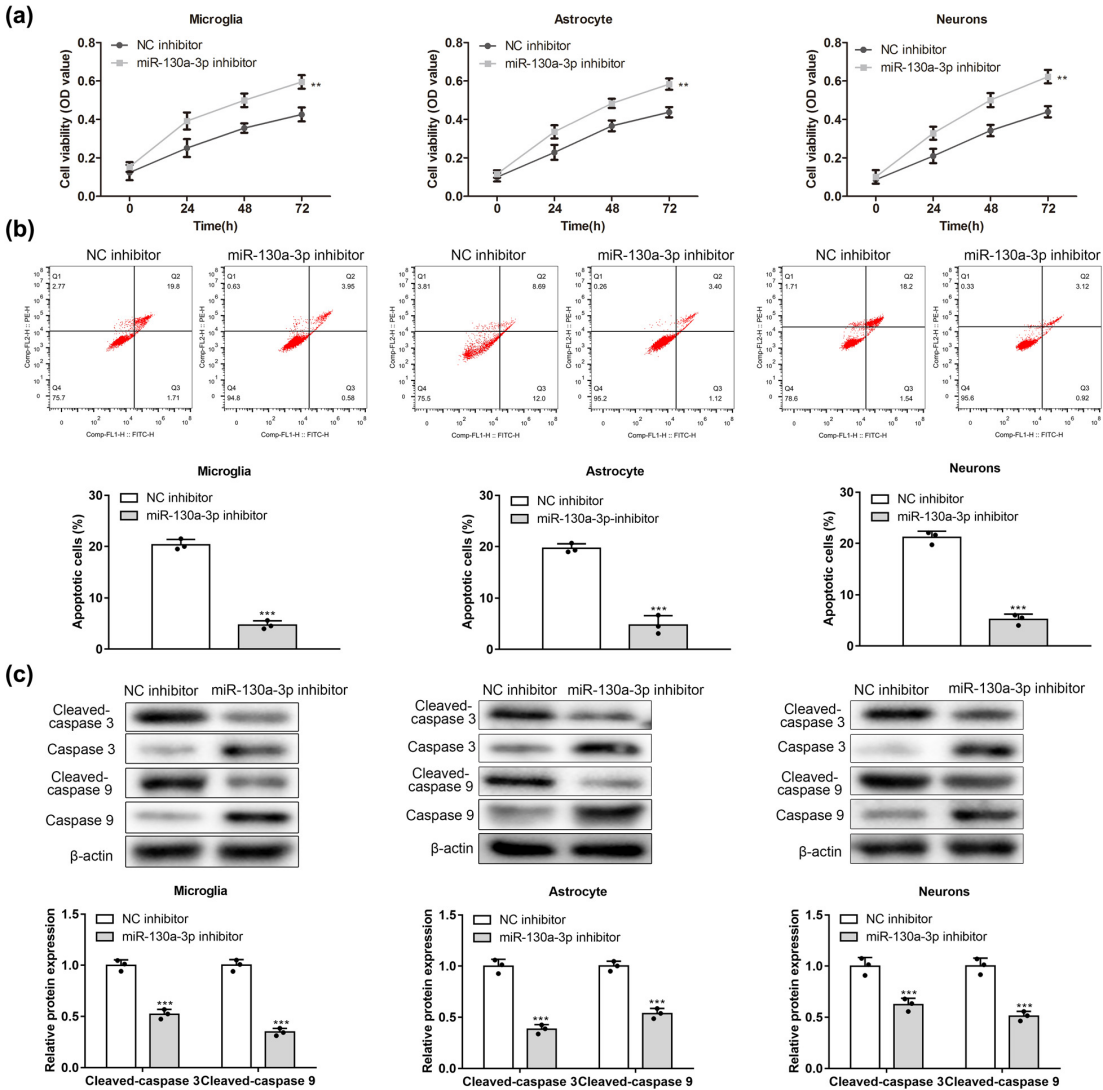
miR-130a-3p inhibitor promoted viability and suppressed apoptosis of microglia, astrocytes, and neurons under the HG condition. (a) Viability of HG-stimulated microglia, astrocytes, and neurons under the transfection of miR-130a-3p inhibitor and NC inhibitor was assessed by CCK-8 assay. (b) Impacts of miR-130a-3p inhibitor on HG-induced apoptosis of cells were analyzed by flow cytometry. (c) Effects of miR-130a-3p inhibitor on cleaved caspase 3/9 proteins in HG-treated cells were evaluated by western blotting. **P < 0.01, ***P < 0.001 vs NC inhibitor.

According to the result of the CCK-8 assay, primary neurons, microglia, and astrocytes showed enhanced viability by treatment of miR-130a-3p inhibitor (Figure 2a). According to Annexin V staining, compared with microglial cells, astrocytes, and neurons by treatment of NC inhibitor, cells with miR-130a-3p inhibitor showed a decreased apoptosis rate (Figure 2b). Protein levels of apoptosis markers, cleaved caspase 3/9, were reduced by miR-130a-3p deficiency (Figure 2c). These results suggest that miR-130a-3p can participate in and regulate the status of primary neurons, microglia, and astrocytes.

### 3.3 MiR-130a-3p deficiency suppressed oxidative stress of neurons under the HG condition

**Figure 3:**
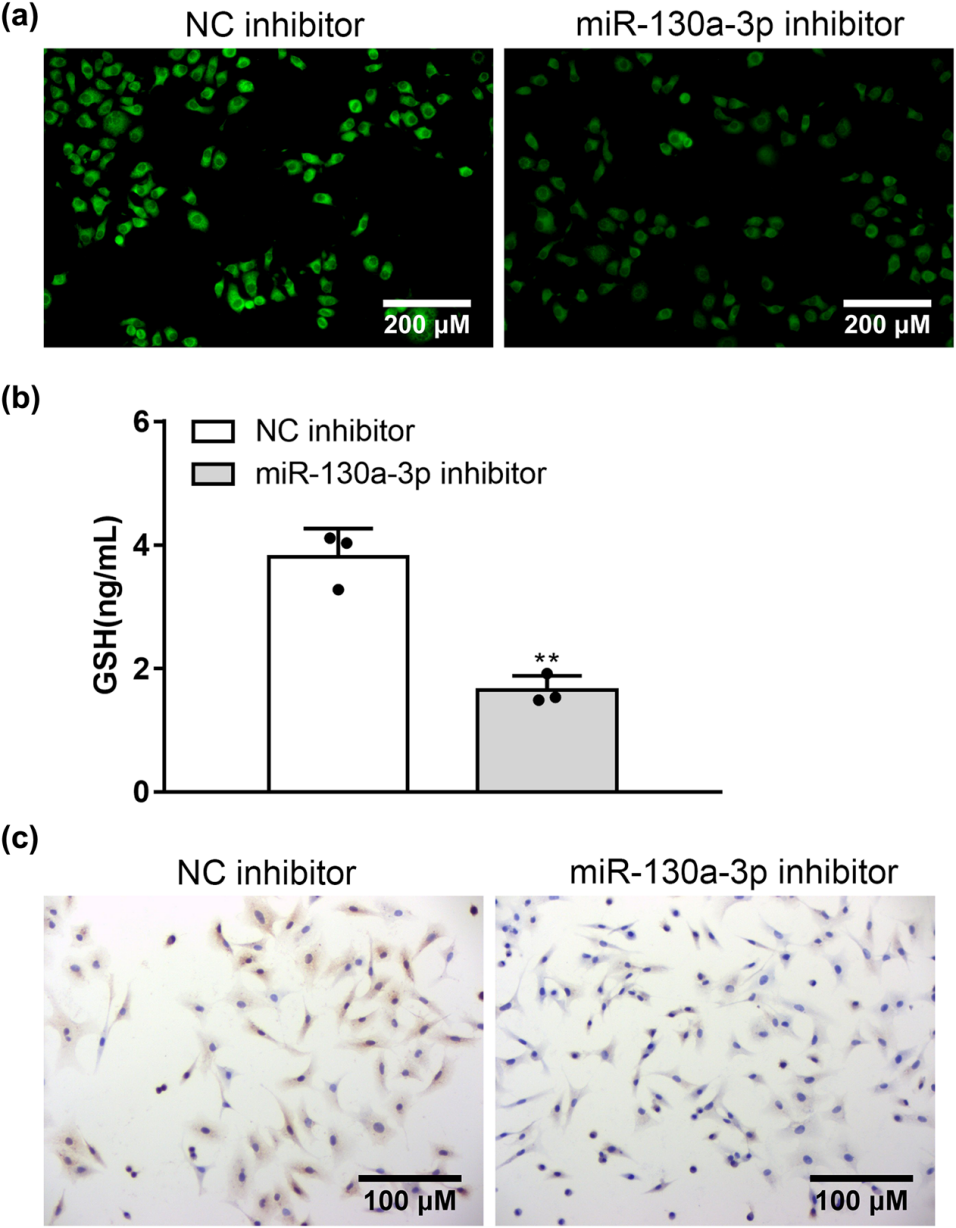
miR-130a-3p inhibitor suppressed oxidative stress of neurons under the HG condition. (a) Impacts of miR-130a-3p inhibitor on ROS level of HG-treated neurons were detected by DCF probe using a commercial kit (bar = 200 μm). (b) Impacts of miR-130a-3p inhibitor on the GSH level of HG-treated neurons. (c) Photographs show Nav1.8 channel expression in neurons (bar = 100 μm). **P < 0.01 vs NC inhibitor.

The suppressive effects of miR-130a-3p knockdown on ROS (Figure 3a) and GSH (Figure 3b) levels in HG-stimulated neurons. Figure 3c demonstrates that after treatment of miR-130a-3p inhibitor, cells exhibited downregulation of Nav1.8 channel expression. These results suggest that miR-130a-3p can participate in and regulate neuronal oxidative stress.

### 3.4 MiR-130a-3p deficiency inactivated the astrocytes and microglial cells

**Figure 4:**
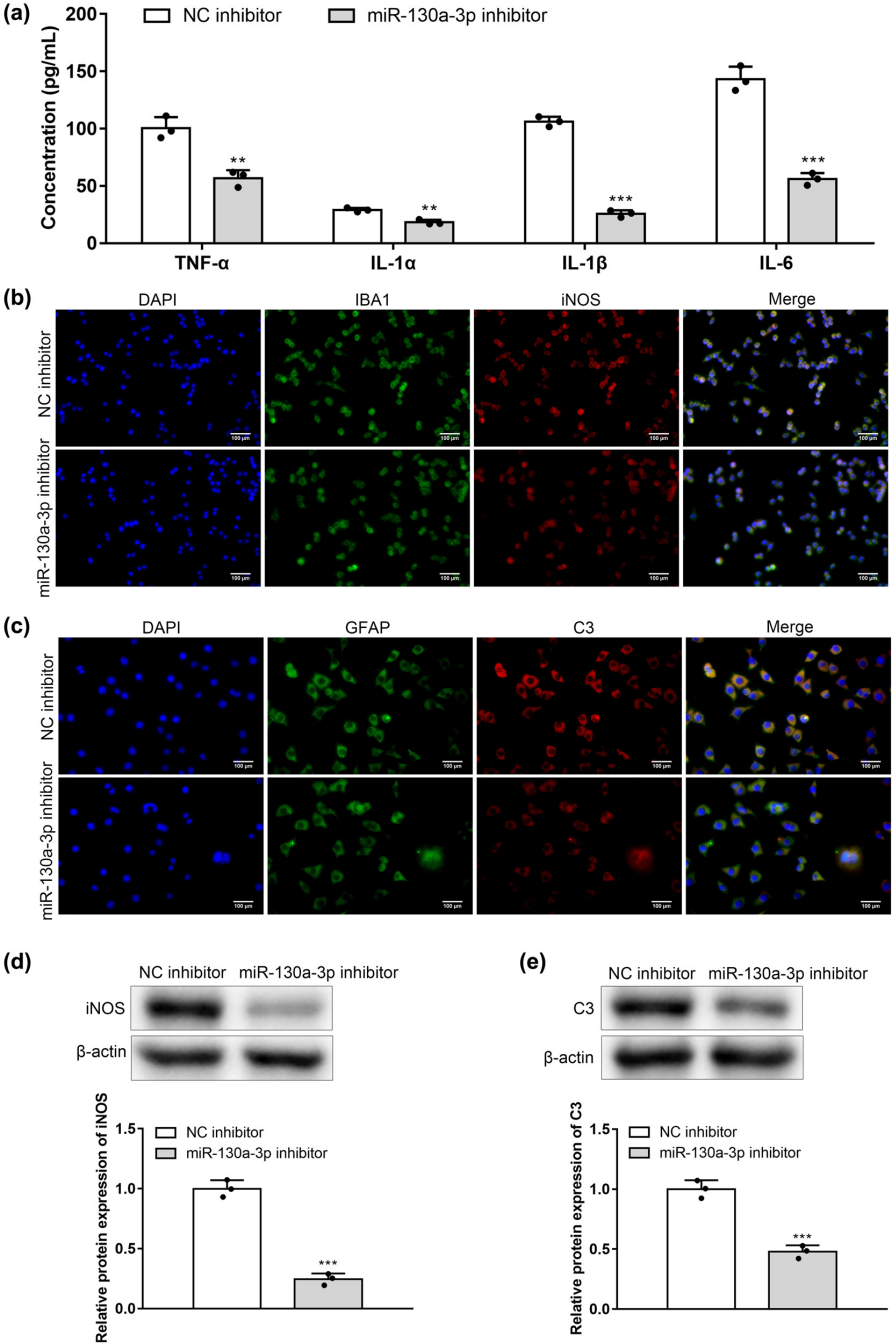
miR-130a-3p deficiency inactivated the astrocytes and microglial cells. (a) Concentrations of IL-1α, IL-1β, TNF-α, and IL-6 in supernatants of microglial cells with miR-130a-3p inhibitor were assessed by ELISA. (b) Immunofluorescence staining of IBA1 and iNOS in microglial cells with miR-130a-3p inhibitor. (c) Immunofluorescence staining of GFAP and C3 in astrocytes with miR-130a-3p inhibitor. (d) Western blotting of iNOS protein in microglial cells. (e) C3 protein in astrocytes with miR-130a-3p inhibitor. **P < 0.01, ***P < 0.001 vs NC inhibitor.

Contents of proinflammatory factors, IL-1α, IL-1β, TNF-α, and IL-6, in microglial cells under the transfection of miR-130a-3p inhibitor were detected by ELISA. MiR-130a-3p deficiency reduced the concentrations of these proinflammatory cytokines (IL-1α, IL-1β, TNF-α, and IL-6) in supernatants of microglial cells (Figure 4a). GFAP and IBA1 are the markers for the astrocytes and microglial cells. iNOS is associated with M1 microglia, and C3 is associated with A1 astrocytes. Based on the results of the immunofluorescence staining assay, silencing of miR-130a-3p decreased IBA1 and iNOS levels in microglial cells (Figure 4b) and GFAP and C3 levels in astrocytes (Figure 4c). MiR-130a-3p inhibitor reduced iNOS protein level in microglial cells (Figure 4d) and C3 protein level in astrocytes (Figure 4e). These results suggest that low expression of miR-130a-3p can reduce the level of inflammation and inactivate microglial cells.

### 3.5 S1PR1 is downstream miR-130a-3p and regulated by it

**Figure 5:**
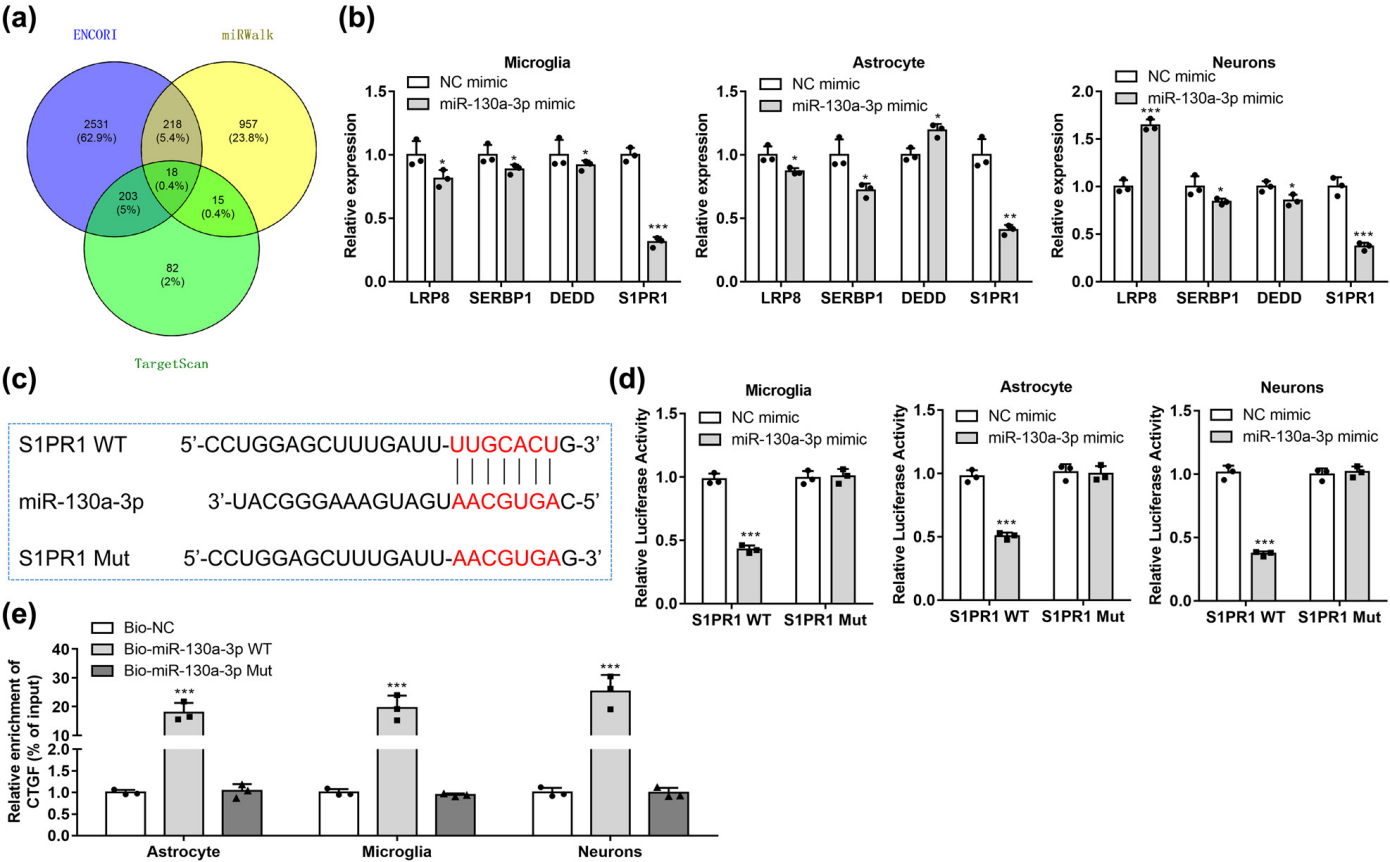
S1PR1 is a target of miR-130a-3p. (a) The Venn diagram shows 18 targeted mRNAs of miR-130a-3p that are predicted by ENCORI, TargetScan, and miRWalk databases. (b) Expression of LRP8, SIPR1, SERBP1, and DEDD was determined by RT-qPCR in microglia, astrocytes, and neurons transfected with miR-130a-3p mimics. (c) Binding sequences between miR-130a-3p and SIPR1 3′-UTR were obtained from TargetScan. (d) A dual luciferase reporter assay for confirmation of the interaction between miR-130a-3p and SIPR1 3′-UTR in microglia, astrocytes, and neurons. (e) RNA pull-down assay was used to verify the relationship between S1PR1 and miR-130a-3p. *P < 0.05, **P < 0.01, ***P < 0.001 vs NC mimic group.

As shown in the Venn diagram, 18 mRNAs are predicted by ENCORI, TargetScan, and miRWalk databases to be targeted by miR-130a-3p (Figure 5a). Among these 18 candidates, LRP8, SERBP1, DEDD, and SIPR1 expression was significantly affected by miR-130a-3p. MiR-130a-3p mimic caused the most significant downregulation of S1PR1 (Figure 5b). The binding site of position 259–265 of S1PR1 3′-UTR on miR-130a-3p was obtained from TargetScan (Figure 5c). Compared with NC mimics, miR-130a-3p mimics negatively mediated pGL3-S1PR1 3′-UTR-WT luciferase activity and had no significant effects on pGL3-S1PR1 3′-UTR-MUT, indicating the binding of S1PR1 and miR-130a-3p at the predicted site (Figure 5d). The results of pull-down experiments showed that biotinylated S1PR1-WT had higher expression levels of miR-130a-3p than S1PR1-Mut or NC groups (Figure 5e). This suggests that S1PR1 is a downstream target gene of miR-130a-3p.

### 3.6 The rescue effects of silenced S1PR1 on silenced miR-130a-3p in apoptosis and oxidative stress of the HG-induced neurons

**Figure 6:**
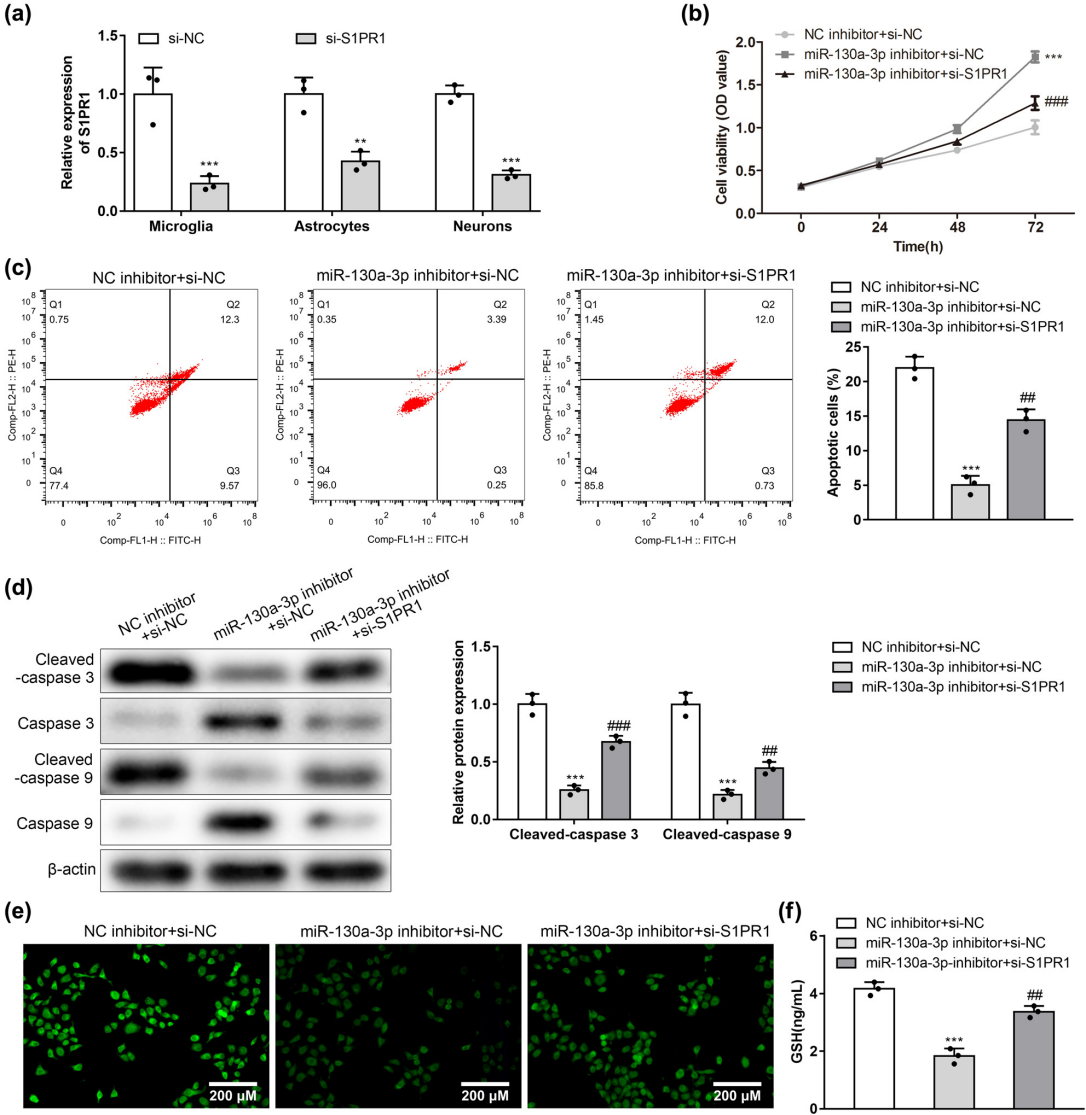
Rescue effects of silenced S1PR1 on silenced miR-130a-3p in apoptosis and oxidative stress of the HG-induced neurons. (a) The expression of S1PR1 was assessed in microglia, astrocytes, and neurons transfected with si-S1PR1 by RT-qPCR. (b) Viability of HG-stimulated neurons under the transfection of miR-130a-3p inhibitor or cotransfection of miR-130a-3p inhibitor + si-S1PR1 was determined by a CCK-8 kit. (c) Flow cytometry apoptosis analysis of HG-stimulated neurons. (d) Rescue effects of si-S1PR1 on miR-130a-3p inhibitor in cleaved caspase 3/9 proteins in HG-treated neurons, as revealed by western blotting. Rescue effects of si-S1PR1 on miR-130a-3p inhibitor in ROS (e) and GSH (f) levels. ***P < 0.001 vs NC inhibitor + si-NC; ^##^ *P* < 0.01, ^###^ *P* < 0.001 vs miR-130a-3p inhibitor + si-NC.

To detect the transfection efficiency of si-S1PR1 in microglia, astrocytes, and neurons. We detected the expression level of S1PR1 in microglia, astrocytes, and neurons by RT-qPCR. The results showed that the expression level of S1PR1 in microglia, astrocytes, and neurons transfected with si-S1PR1 decreased significantly (Figure 6a). The miR-130a-3p knockdown-induced increase in neural viability and decrease in neural apoptosis rate was partially restored by silencing of S1PR1 (Figure 6b and c). Cleaved caspase 3/9 protein levels were repressed under miR-130a-3p deficiency. Such effect was rescued by the S1PR1 knockdown (Figure 6d). Moreover, silenced S1PR1 rescued the suppressive effects of miR-130a-3p knockdown on ROS and GSH levels (Figure 6e and f). This suggests that miR-130a-3p can regulate neuronal apoptosis and oxidative stress through S1PR1.

### 3.7 si-S1PR1 rescued the repressive effects of the miR-130a-3p inhibitor on activation of the astrocytes and microglial cells

**Figure 7:**
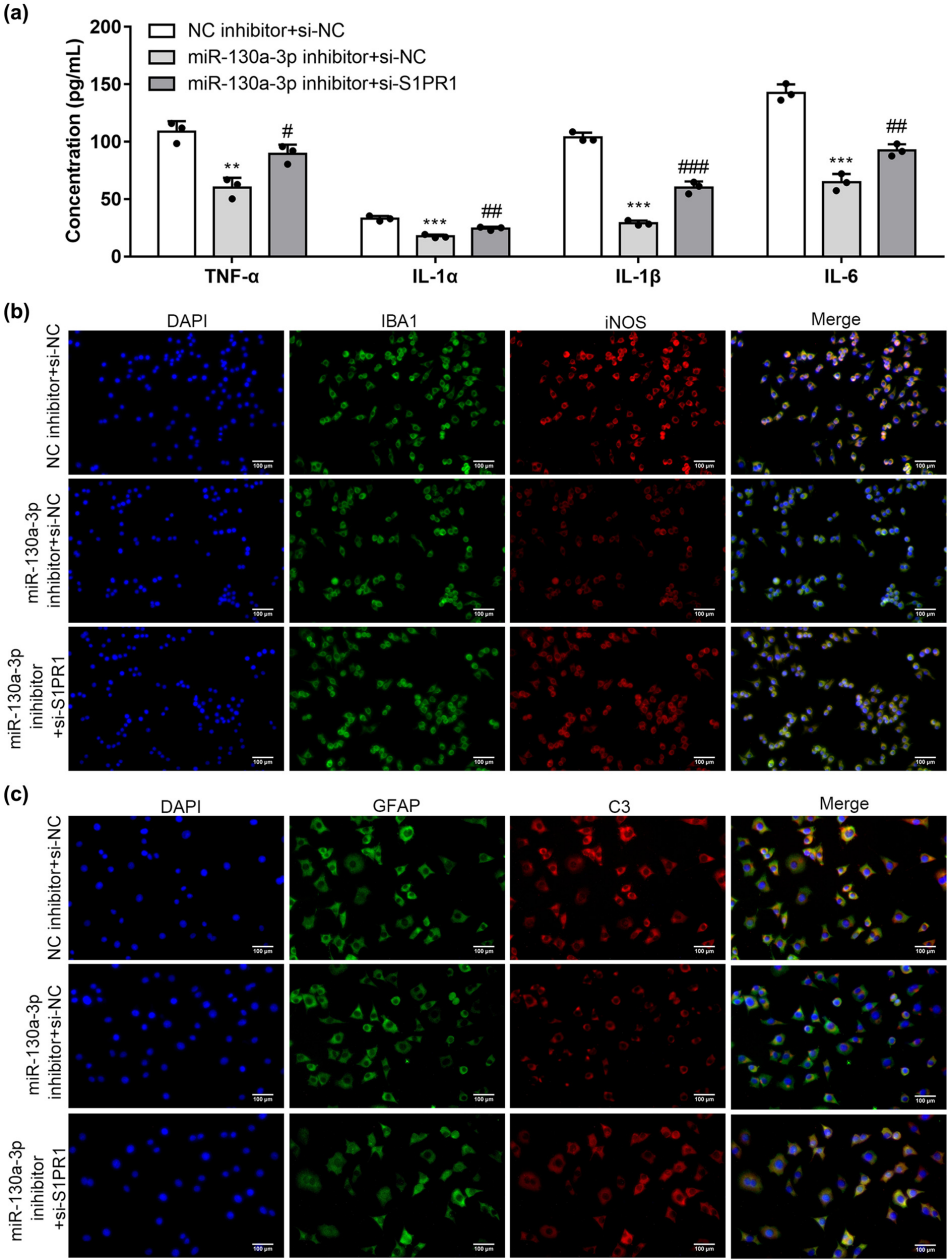
si-S1PR1 rescued the repressive effects of the miR-130a-3p inhibitor on activation of the astrocytes and microglial cells. (a) Rescue effects of S1PR1 deficiency on miR-130a-3p deficiency in IL-1α, TNF-α, IL-6, and IL-1β concentrations in supernatants of microglia were assessed by ELISA. (b) Immunofluorescence staining of IBA1 and iNOS in microglial cells under the transfection of miR-130a-3p inhibitor or cotransfection of miR-130a-3p inhibitor + si-S1PR1. (c) Immunofluorescence staining of GFAP and C3 in astrocytes by treatment of miR-130a-3p inhibitor or cotransfection with miR-130a-3p inhibitor + si-S1PR1. ***P < 0.001 vs NC inhibitor + si-NC, ^#^ *P* < 0.05, ^##^ *P* < 0.01, ^###^ *P* < 0.001 vs miR-130a-3p inhibitor + si-NC.

As revealed by the results of ELISA in Figure 7a, S1PR1 knockdown rescued the miR-130a-3p inhibitor-induced decrease in concentrations of proinflammatory factors. Figure 7b and c reveals that the miR-130a-3p deficiency-induced decrease in IBA1 and iNOS levels in microglial cells and GFAP and C3 levels in astrocytes were rescued by si-S1PR1, which suggested that miR-130a-3p activated the microglial cells and astrocytes by targeting S1PR1. This suggests that miR-130a-3p can regulate astrocyte and microglial activation through S1PR1.

## 4 Discussion

MiR-130a is upregulated in rats with type-2 diabetes and has diagnostic value for this disease [30,31]. Zhang et al. found that miR-130a promotes neuronal growth and attenuates neuronal injury after ICH in rat brains through the PTEN/PI3K/Akt signaling pathway [32]. Zhang et al. found that inhibition of miR-130a-3p significantly reduced NP symptoms, attenuated neuronal apoptosis, microglial activation, and suppressed inflammation in SCI rats [25]. We used the HG-stimulated neurons to mimic the diabetes-induced neuron injury and found that HG induced the upregulation of miR-130a-3p in neurons, which indicated the putative role of miR-130a-3p in DPN. MiR-130a-3p silence decreased the apoptosis rate of neurons and reduced the proteins of the apoptotic pathway. MiR-130a-3p knockdown reduced the oxidative stress by decreasing ROS and GSH levels, and suppressing Nav1.8 channel expression in neurons under the HG stimulation.

Moreover, it was demonstrated that miR-130a-3p was upregulated in HG-stimulated microglial and astrocytes. MiR-130a-3p inhibition can reduce the spinal cord injury-induced NP symptoms in rats and mitigate microglial activation [25]. Overexpression of miR-130a-5p can relieve NP in mice with astrocyte activation [33]. The present study revealed that miR-130a-3p silencing reduced the activation of microglia and astrocytes, suppressing cell apoptosis *in vitro*. All these findings revealed that targeting miR-130a-3p may be beneficial for DPN treatment by suppressing neurodegeneration and neuroinflammation.

Subsequently, based on bioinformatics analysis, SIPR1 was demonstrated as a target downstream miR-130a-3p. MiR-130a-3p-induced downregulation of SIPR1 via binding to SIPR1 3′-UTR. The direct interaction between miR-130a-3p and SIPR1 3′-UTR was also confirmed by Chen et al. [34].

S1P is related to excitability, growth, survival, and apoptosis in neurons [35]. SIPR1 exists in afferent sensory neurons [36] and regulates nociceptor function via the intracellular pathways [37]. S1P induces acute pain by binding to SIPR1 [37]. The rise of S1P levels in neurons is accompanied by increased microglial activation [38]. Microglia cells express all S1P receptors, including SIPR1, and expression of the S1P receptor is closely associated with microglia activation [39]. SIPR1 has been indicated to be downregulated in activated microglia [40], and SIPR1 deficiency in microglia suppresses microglial activation [41]. We found that SIPR1 knockdown promoted the activation of microglia cells and astrocytes, positively regulating apoptosis and oxidative stress of the HG-stimulated neurons, indicating the protective role of SIPR1 in DPN.

In conclusion, we revealed the significant role of miR-130a-3p inhibition in the alleviation of neuronal injury, microglial, and astrocyte activation by its target SIPR1, which indicated that targeting miR-130a-3p can be developed as the therapeutic method for DPN treatment. However, the limitations of our study must be stated. The major drawback is the lack of the *in vivo* model of DPN. Second, other potential genes that exert functions alone or together with miR-130a-3p cannot be ruled out. More studies will be conducted in the future to overcome these limitations.

## References

[1] Crofford OB. Diabetes control and complications. Annu Rev Med. 1995;46:267–79. 10.1146/annurev.med.46.1.267. 7598463

[2] Obrosova IG. Diabetic painful and insensate neuropathy: pathogenesis and potential treatments. Neurotherapeutics. 2009;6(4):638–47. 10.1016/j.nurt.2009.07.004. PMC5084286 19789069

[3] Ziegler D. Painful diabetic neuropathy: treatment and future aspects. Diabetes Metab Res Rev. 2008;24(Suppl 1):S52–7. 10.1002/dmrr.817. 18395890

[4] Molines L, Darmon P, Raccah D. Charcot’s foot: newest findings on its pathophysiology, diagnosis and treatment. Diabetes Metab. 2010;36(4):251–5. 10.1016/j.diabet.2010.04.002. 20570543

[5] Pop-Busui R, Sima A, Stevens M. Diabetic neuropathy and oxidative stress. Diabetes Metab Res Rev. 2006;22(4):257–73. 10.1002/dmrr.625. 16506271

[6] McMahon SB, Cafferty WB, Marchand F. Immune and glial cell factors as pain mediators and modulators. Exp Neurol. 2005;192(2):444–62. 10.1016/j.expneurol.2004.11.001. 15755561

[7] Biggs JE, Lu VB, Stebbing MJ, Balasubramanyan S, Smith PA. Is BDNF sufficient for information transfer between microglia and dorsal horn neurons during the onset of central sensitization. Mol Pain. 2010;6:44. 10.1186/1744-8069-6-44. PMC2918544 20653959

[8] Scholz J, Woolf CJ. The neuropathic pain triad: neurons, immune cells and glia. Nat Neurosci. 2007;10(11):1361–8. 10.1038/nn1992. 17965656

[9] Budnik V, Ruiz-Cañada C, Wendler F. Extracellular vesicles round off communication in the nervous system. Nat Rev Neurosci. 2016;17(3):160–72. 10.1038/nrn.2015.29. PMC4989863 26891626

[10] Wang D, Couture R, Hong Y. Activated microglia in the spinal cord underlies diabetic neuropathic pain. Eur J Pharmacol. 2014;728:59–66. 10.1016/j.ejphar.2014.01.057. 24508519

[11] Ismail CAN, Suppian R, Ab Aziz CB, Long I. Expressions of spinal microglia activation, BDNF, and DREAM proteins correlated with formalin-induced nociceptive responses in painful and painless diabetic neuropathy rats. Neuropeptides. 2020;79:102003. 10.1016/j.npep.2019.102003. 31902597

[12] Mracsko E, Veltkamp R. Neuroinflammation after intracerebral hemorrhage. Front Cell Neurosci. 2014;8:388. 10.3389/fncel.2014.00388. PMC4238323 25477782

[13] Kanazawa M, Ninomiya I, Hatakeyama M, Takahashi T, Shimohata T. Microglia and monocytes/macrophages polarization reveal novel therapeutic mechanism against stroke. Int J Mol Sci. 2017;18(10):2135. 10.3390/ijms18102135. PMC5666817 29027964

[14] Orihuela R, McPherson CA, Harry GJ. Microglial M1/M2 polarization and metabolic states. Br J Pharmacol. 2016;173(4):649–65. 10.1111/bph.13139. PMC4742299 25800044

[15] Zhao H, Garton T, Keep RF, Hua Y, Xi G. Microglia/macrophage polarization after experimental intracerebral hemorrhage. Transl Stroke Res. 2015;6(6):407–9. 10.1007/s12975-015-0428-4. PMC4628553 26446073

[16] Tokarz F, Stachowski B. Injury of the vertebral column and cervical medulla complicated by circulatory insufficiency of vertebral arteries. Patol Pol. 1974;25(3):445–9. 4431630

[17] Falzi M, Buscaglia GM. The leprotic foot. Fracastoro. 1969;62(2):207–10. 5404890

[18] Reichenbach N, Delekate A, Plescher M, Schmitt F, Krauss S, Blank N, et al. Inhibition of Stat3-mediated astrogliosis ameliorates pathology in an Alzheimer’s disease model. EMBO Mol Med. 2019;11(2):e9665. 10.15252/emmm.201809665. PMC6365929 30617153

[19] Prabodha LBL, Sirisena ND, Dissanayake VHW. Susceptible and prognostic genetic factors associated with diabetic peripheral neuropathy: a comprehensive literature review. Int J Endocrinol. 2018;2018:8641942. 10.1155/2018/8641942. PMC5875044 29736170

[20] Friedman RC, Farh KK, Burge CB, Bartel DP. Most mammalian mRNAs are conserved targets of microRNAs. Genome Res. 2009;19(1):92–105. 10.1101/gr.082701.108. PMC2612969 18955434

[21] Zhou Y, Wang Z, Chen X, Zhang J, Yang L, Liu S, et al. Identification of differentially expressed miRNAs and mRNAs in synovial of osteoarthritis via RNA-sequencing. BMC Med Genet. 2020;21(1):46. 10.1186/s12881-020-0978-5. PMC7053084 32122327

[22] Jiang BC, Cao DL, Zhang X, Zhang ZJ, He LN, Li CH, et al. CXCL13 drives spinal astrocyte activation and neuropathic pain via CXCR5. J Clin Invest. 2016;126(2):745–61. 10.1172/jci81950. PMC4731172 26752644

[23] Dai X, Cai Z, Liu J. Up-regulation of miR-338-5p after spinal cord injury enhances the neuronal repair via inhibition of inflammation aggravation and oxidative stress. Minerva Med. 2021;112(4):533–4. 10.23736/s0026-4806.19.06280-3. 31833735

[24] Ciccacci C, Latini A, Colantuono A, Politi C, D’Amato C, Greco C, et al. Expression study of candidate miRNAs and evaluation of their potential use as biomarkers of diabetic neuropathy. Epigenomics. 2020;12(7):575–85. 10.2217/epi-2019-0242. 32400192

[25] Yao L, Guo Y, Wang L, Li G, Qian X, Zhang J, et al. Knockdown of miR-130a-3p alleviates spinal cord injury induced neuropathic pain by activating IGF-1/IGF-1R pathway. J Neuroimmunol. 2021;351:577458. 10.1016/j.jneuroim.2020.577458. 33360969

[26] Periyasamy P, Liao K, Kook YH, Niu F, Callen SE, Guo ML, et al. Cocaine-mediated downregulation of miR-124 activates microglia by targeting KLF4 and TLR4 signaling. Mol Neurobiol. 2018;55(4):3196–210. 10.1007/s12035-017-0584-5. PMC5673594 28478506

[27] Ryu KY, Lee HJ, Woo H, Kang RJ, Han KM, Park H, et al. Dasatinib regulates LPS-induced microglial and astrocytic neuroinflammatory responses by inhibiting AKT/STAT3 signaling. J Neuroinflammation. 2019;16(1):190. 10.1186/s12974-019-1561-x. PMC6815018 31655606

[28] Sharma D, Singh JN, Sharma SS. Effects of 4-phenyl butyric acid on high glucose-induced alterations in dorsal root ganglion neurons. Neurosci Lett. 2016;635:83–9. 10.1016/j.neulet.2016.10.038. 27777138

[29] Ippolito DM, Eroglu C. Quantifying synapses: an immunocytochemistry-based assay to quantify synapse number. J Vis Exp. 2010##45)). 10.3791/2270. PMC3159596 21113117

[30] Esguerra JL, Bolmeson C, Cilio CM, Eliasson L. Differential glucose-regulation of microRNAs in pancreatic islets of non-obese type 2 diabetes model Goto-Kakizaki rat. PLoS One. 2011;6(4):e18613. 10.1371/journal.pone.0018613. PMC3072418 21490936

[31] Yan LN, Zhang X, Xu F, Fan YY, Ge B, Guo H, et al. Four-microRNA signature for detection of type 2 diabetes. World J Clin Cases. 2020;8(10):1923–31. 10.12998/wjcc.v8.i10.1923. PMC7262691 32518782

[32] Zhang CY, Ren XM, Li HB, Wei W, Wang KX, Li YM, et al. Effect of miR-130a on neuronal injury in rats with intracranial hemorrhage through PTEN/PI3K/AKT signaling pathway. Eur Rev Med Pharmacol Sci. 2019;23(11):4890–7. 10.26355/eurrev_201906_18077. 31210323

[33] Dong J, Xu C, Xia R, Zhang Z. Upregulating miR-130a-5p relieves astrocyte over activation-induced neuropathic pain through targeting C-X-C motif chemokine receptor 12/C-X-C motif chemokine receptor 4 axis. Neuroreport. 2021;32(2):135–43. 10.1097/wnr.0000000000001573. 33395188

[34] Chen X, Zhang J, Liu Z, Zhang S, Sun T. Specific microRNA signatures responsible for immune disturbance related to hip fracture in aged rats. J Orthop Surg Res. 2018;13(1):17. 10.1186/s13018-018-0721-5. PMC5778820 29357879

[35] Okada T, Kajimoto T, Jahangeer S, Nakamura S. Sphingosine kinase/sphingosine 1-phosphate signalling in central nervous system. Cell Signal. 2009;21(1):7–13. 10.1016/j.cellsig.2008.07.011. 18694820

[36] Chi XX, Nicol GD. The sphingosine 1-phosphate receptor, S1PR₁, plays a prominent but not exclusive role in enhancing the excitability of sensory neurons. J Neurophysiol. 2010;104(5):2741–8. 10.1152/jn.00709.2010. PMC2997035 20844107

[37] Mair N, Benetti C, Andratsch M, Leitner MG, Constantin CE, Camprubí-Robles M, et al. Genetic evidence for involvement of neuronally expressed S1P₁ receptor in nociceptor sensitization and inflammatory pain. PLoS One. 2011;6(2):e17268. 10.1371/journal.pone.0017268. PMC3040773 21359147

[38] Karunakaran I, Alam S, Jayagopi S, Frohberger SJ, Hansen JN, Kuehlwein J, et al. Neural sphingosine 1-phosphate accumulation activates microglia and links impaired autophagy and inflammation. Glia. 2019;67(10):1859–72. 10.1002/glia.23663. 31231866

[39] Noda H, Takeuchi H, Mizuno T, Suzumura A. Fingolimod phosphate promotes the neuroprotective effects of microglia. J Neuroimmunol. 2013;256(1–2):13–8. 10.1016/j.jneuroim.2012.12.005. 23290828

[40] Tham CS, Lin FF, Rao TS, Yu N, Webb M. Microglial activation state and lysophospholipid acid receptor expression. Int J Dev Neurosci. 2003;21(8):431–43. 10.1016/j.ijdevneu.2003.09.003. 14659994

[41] Choi JW, Gardell SE, Herr DR, Rivera R, Lee CW, Noguchi K, et al. FTY720 (fingolimod) efficacy in an animal model of multiple sclerosis requires astrocyte sphingosine 1-phosphate receptor 1 (S1P1) modulation. Proc Natl Acad Sci U S A. 2011;108(2):751–6. 10.1073/pnas.1014154108. PMC3021041 21177428

